# Legacies to Hospitals

**Published:** 1911-03-25

**Authors:** 


### LEGACIES TO HOSPITALS.

The case of Wallace *v.* Mawdsley, which was decided by Mr. Justice Joyce last week, brings to light a strange example of a mistake on the part of a charitable testatrix, which had the effect of defeating the object she had in view. When drawing up her will, with the assistance of her solicitor, she arranged to divide her residuary estate amongst certain hospitals and institutions in Liverpool, including the Deaf and Dumb Asylum, Liverpool, and Dr. Barnardo's Homes. Her solicitor then pointed out that she had given nothing to any hospital for women, and he accordingly suggested and recommended to her a certain hospital as a proper object of her bounty if she were disposed to make such a bequest. Accordingly, " The Royal Hospital for Women " was mentioned in the will as a participator in the residuary estate. When the time arrived for distributing the bequest it appeared that there was no institution answering the description of " The Deaf and Dumb Asylum, Liverpool." Two institutions, namely, the School for Deaf and Dumb, Oxford Street, Liverpool, and the Liverpool Adult Deaf and Dumb Benevolent Society, laid claim to it. Mr. Justice Joyce decided that each should be admitted to a half-share. A more interesting question arose as to the bequest to the Royal Hospital for Women. There is no such institution, but claim was laid to the fund by the following: The Royal Waterloo Hospital for Women and Children; the Chelsea Hospital for Women; the Hospital for Women, Soho; Hospital for Women, Liverpool; New Hospital for Women; and the Royal Free An attempt was made, however, to ask the judge to accept parole evidence of what took place when the solicitor was receiving instructions to prepare the will. He was ready to prove that the institution which the lady had in mind was the Chelsea Hospital for Women; but Mr. Justice Joyce turned a deaf ear to this evidence. In the event, the question not being capable of decision in favour of any particular institution, it was left for the Master in Chambers to settle a scheme for the disposal of this share of the residuary estate. It always seems ungracious to deplore the carelessness of a generous benefactor; but it generally happens in cases such as this, that before the fact of an attempted gift is made known to the intended beneficiary, the testator or testatrix is beyond the reach of human criticism. It is, however, important that cases of this kind should occasionally be thrashed out in court, for they draw attention to the results of carelessness in making a will. In the present instance no fewer than six firms of solicitors and fifteen gentlemen of the long robe were engaged in trying to unravel the knot; and, as the knot was tied by the generous lady herself, it is presumed that the trifle of costs incurred will come out of the estate. If we were to re-draft the maxim, " You must not look a gift horse in the mouth," we should be inclined to add the proviso: " provided always that this maxim shall not extend to prevent the managers of any hospital from making known to a testator the exact name and address of such hospital."

